# Clinical Epidemiology of Head Injury from Road-Traffic Trauma in a Developing Country in the Current Era

**DOI:** 10.3389/fneur.2017.00695

**Published:** 2017-12-15

**Authors:** Amos O. Adeleye, Millicent I. Ogun

**Affiliations:** ^1^Division of Neurological Surgery, Department of Surgery, College of Medicine, University of Ibadan, Ibadan, Nigeria; ^2^Department of Neurological Surgery, University College Hospital (UCH), Ibadan, Nigeria; ^3^Clinical Nursing Department, University College Hospital (UCH), Ibadan, Nigeria

**Keywords:** road-traffic injury, head injury, motor-vehicle crash, motorcycle crash, Nigeria, developing countries

## Abstract

**Objectives:**

Africa and other Asian low middle-income countries account for the greatest burden of the global road-traffic injury (RTI)-related head injury (HI). This study set out to describe the incidence, causation, and severity of RTI-related HI and associated injuries in a Nigerian academic neurosurgical practice.

**Methods:**

This is a retrospective cross-sectional analysis of RTI-related HI from a prospective HI registry in an academic neurosurgery practice in Nigeria.

**Results:**

All-terrain RTI accounted for 80.6% (833/1,034) of HI over a 7-year study period. All age groups were involved, mean 33.06 years (SD 18.30), mode 21–30, 231/833 (27.7%). The male:female ratio was 631:202, ≈3:1. The road trauma occurred exclusively from motorcycle-and motor-vehicle crash (MCC/MVC), MCC caused 56.8% (473/833) of these; the victims were vulnerable road users (VRU) in 74%, and >90% belong in the low socioeconomic class. Using the Glasgow Coma Scale grading, the HI was moderate/severe in 52%; loss of consciousness occurred in 93%, the Abbreviated Injury Severity-head > 3 in 74%, and computed tomography (CT) Rotterdam score > 3 in 52%. Significant extracranial injuries occurred in many organ systems, 421/833 (50.5%) having Injury Severity Score (ISS) > 25. Surgical lesions included extensive brain contusions in 157 (18.8%); acute extradural hematoma in 34 (4.1%); acute subdural hematoma in 32 (3.8%); and traumatic intracerebral hemorrhage in 27 (3.2%), but only 97 (11.6%) received operative care for various logistic reasons. The in-hospital outcome was good in 71.3% and poor in 28.7%; the statistically significant (*p* < 0.001) determinants of this outcome profile were the severity of the HI, the CT Rotterdam score, and the ISS.

**Conclusion:**

In this study from Nigeria, RTI-related HI emanates from significant trauma to vulnerable road users and are caused exclusively by motorcycles and motor vehicles.

## Introduction

Head injury (HI) usually accounts for the major proportion of case fatalities in studies that examine the burden of road-traffic injuries (RTI), also known as road-traffic accidents (1–4). Although a global phenomenon, RTI burdens in general, and RTI-related fatalities in particular, exert their highest tolls in the low middle-income countries (LMIC) of the world (1, 5–7). It is particularly more so in the African region (1, 8–10).

Most previous reports on HI in Africa are dated (11, 12), and are from all etiologies even though in the majority, road trauma was usually the most frequent cause (13–15). In most African countries, increasing motorization in recent times at the same time of rapid deterioration of national road infrastructures (1, 8, 9) invariably translates to a daily deluge of RTI-related cases of HI in most neurosurgical units.

This study is from an academic neurosurgical practice in Nigeria. The aim is to define salient clinical–epidemiological and sociodemographic characteristics of RTI-related HI as seen usually in this large, sub-Sahara African country. This, it is hoped, may aid in the evolution of effectual precautionary measures to reduce the currently rampant RTI burdens in this developing country.

## Materials and Methods

This is a cross-sectional analysis of cases of RTI-related HI managed by a neurosurgeon in a developing country’s university teaching hospital.

### Study Setting

This hospital is a large university teaching hospital, about 1,000 bed in size. It had a four-member neurosurgical faculty in this study period. There were many other neurosurgical units in our country but this hospital is our country’s center of excellence in neurosciences, houses the nation’s premier and foremost neurosurgical program, and is, as such, at the pinnacle of the national referral system for neurosurgical care in general (16).

### Data Collection

The subjects were extracted from a personal prospective HI registry. This data capturing, spanning 82 months in this study (August 2009 till June 2016), was from the patients’ clinical discharge summaries, a paper-based clinical records of patients which consultant physicians have institutional approval to keep in our practice. These were cases of HI managed exclusively by the senior author as a consultant neurosurgeon in our university teaching hospital. Each case’s clinical information, that is their in-hospital course down to their last outpatient follow-up visit post hospital discharge, was conscientiously captured prospectively and consecutively by the senior author as an ongoing personal clinical–academic project. Some of the unit resident doctors gave generous help in this exercise. The database was then transcribed into electronic spread sheet, using the SPSS software (The SPSS Inc., IL, USA). Cases of HI resulting from all-terrain road crashes were then extracted from the database for this study. Information retrieved included age, gender, brief details of the road accidents, clinical findings including the nature and severity of head injuries and extra cranial injuries, time to treatment, radiological examination findings, surgical interventions, and outcomes. The severity of the HI was quantified with the Glasgow Coma Scale (GCS) (17, 18) as well as the Head-Abbreviated Injury Severity (AIS-head) score (1990 version) (17, 19). The GCS 13–15 was categorized mild HI; 9–12 as moderate, and 3–8 as severe HI. Extracranial injuries were also quantified with respective systemic AIS. The trauma severity in each patient was then quantified with the Injury Severity Score (ISS), ranging from 1 to 75, higher number denoting more severe trauma (19). The brain computed tomography (CT) findings were also analyzed and were categorized using the CT Rotterdam (CT Rott) scoring system, quantified on a scale of 1–6 (20). Higher scores represent more significant brain injuries on this scale.

The Glasgow Outcome Scale (GOS) was calculated to determine outcome. The GOS was graded as normal, moderate deficit, severe deficit, persistent vegetative state, and death (19).

### Statistical Analysis

The data were analyzed with the same SPSS statistical software. The findings are presented in sizes, frequencies, and proportions for categorical variables; and the mean (SD) or median (interquartile range, IQR) for continuous variables. The chi-square or Fishers’ exact test was used for associations between categorical variables. These were reported as proportions, unadjusted odds ratio UOR (95% confidence interval, CI). The Student’s *t*-test was used for continuous parametric variables, results expressed as mean (SD), OR (95%CI). The *p*-value < 0.05 was deemed statistically significant. The GOS was dichotomized to categorize the outcome into good (GOS: normal and moderate deficit) and poor (GOS: severe deficit, persistent vegetative state, and death). Other variables were also dichotomized for the purpose of statistical analysis as follows: the ISS (≤25 versus >25); CT Rott score (≤3 versus >3); and AIS-head (≤3 versus >3).

## Results

**Figure 1:**
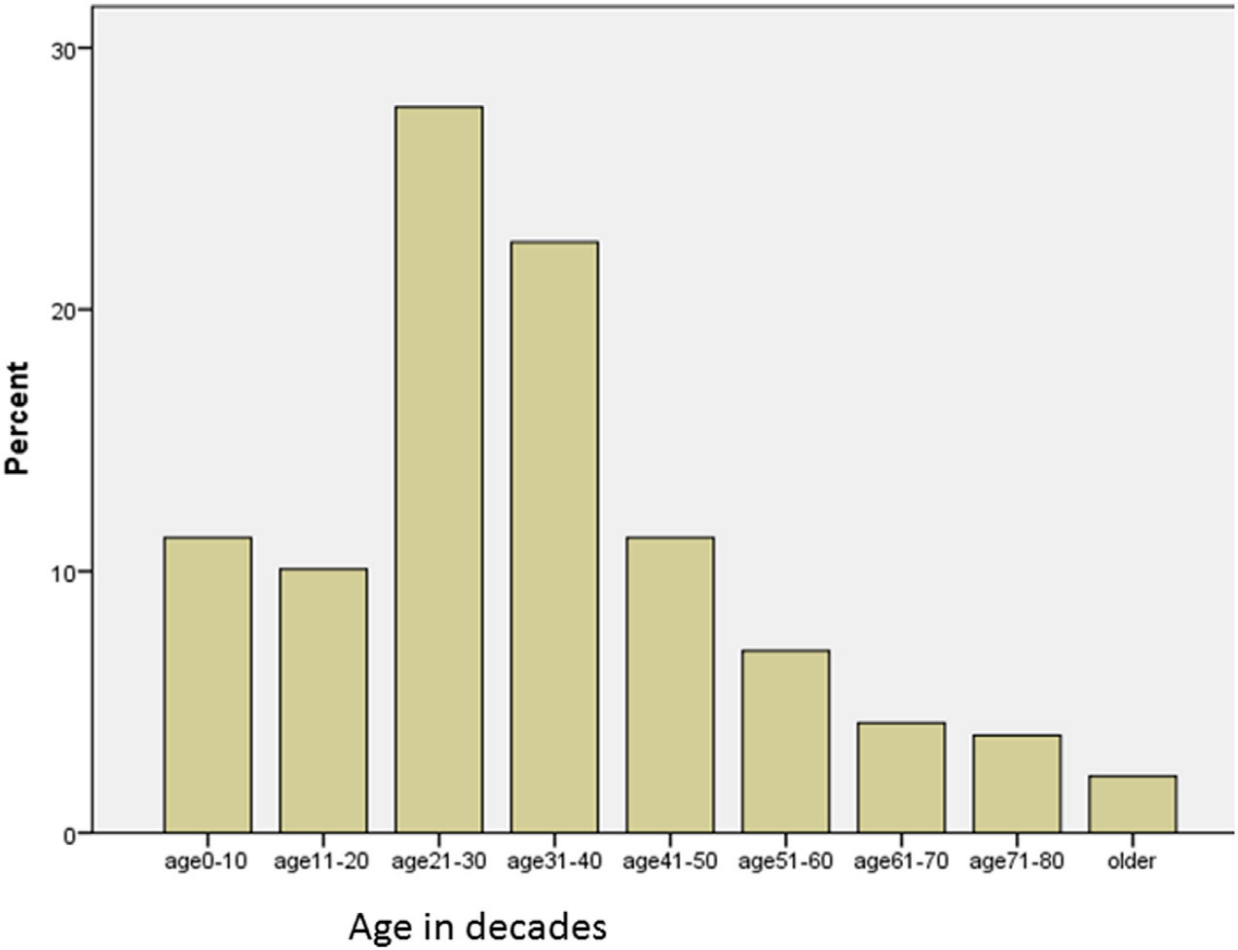
Road trauma-related head injury from Nigeria: age distribution of the victims in this study.

A total of 1,034 cases of HI was recorded in the registry between August 2009 and June 2016. Out of these, 833 (80.6%) were due to all-terrain road accidents. There were 631 males and 202 females, male:female ratio of about 3:1. All age groups were captured, ranging from 1 week to 85 years, mean 33.06 years (SD 18.30). Figure 1 shows the age distribution in decades: the modal age group was the 21–30, accounting for 231/833 (27.7%); 50.3% involved people in the third and fourth decades of life, and people in the first five decades of life accounted for 83% of cases. They were also dependants (students/unemployed) or low socioeconomic class (subsistence traders and low-level civil servants) in 96%.

Table 1 shows more details of this study population’s trauma-related demographics. The road trauma involved only motorized vehicles; motorcycle crash (MCC) accounted for more than half, 473/833 (56.8%) and the victims were pedestrians in about a third, 267/833 (32.1%). In another light, it can be seen that vulnerable road users (VRU), that is, MCC victims plus pedestrian RTI victims, constituted 613/833 (73.6%) of these cohorts. The increased vulnerability of this group stems from the suboptimal road infrastructures and transportation systems of most LMIC. This is seen partly in the lack of separation of these category of road users from fast-moving motorized vehicles in the high-intensity traffic mix of most developing countries (8).

**Table 1:** Road-traffic injury-related head injury from Nigeria: characteristics of the study subjects.

Variable	*N* (%)
**Gender (*N* = 833)**	
Male	631 (75.8)
Female	202 (24.2)

**Occupation (*N* = 789)**	
Unemployed, students	210 (26.6)
Artisans/traders	434 (55.0)
Junior civil servants	112 (14.2)
Senior civil servants/professionals	33 (4.2)

**Cause of road trauma (*N* = 833)**	
Motor vehicles	360 (43.2)
Motor cycles	473 (56.8)

**Categories of trauma victims (*N* = 833)**	
Riders/drivers	254 (30.5)
Passengers	312 (37.5)
Pedestrians	267 (32.0)

### In-Hospital Clinical Findings

Presentation for neurosurgical care was delayed in most cases, the median trauma-to-evaluation time being 14 h (IQR 5.0–48.0). Most cases (93%) suffered loss of consciousness, median duration of 16 h (IQR 3–72). And as shown in Tables 2 and 3, other clinical symptoms and signs of significant trauma/brain injury like vomiting, seizures, anemia, and anisocoria, as well as significant extracranial systemic injuries occurred in notable proportions of the patients. Indeed, ISS > 25 occurred in 421/833 (50.5%); the AIS-head > 3 in 612 (73.5%); and more than 50% suffered moderate–severe HI using the GCS scoring. There were 415/833 (49.8%) mild, 230 (27.6%) moderate, and 188 (22.6%) severe HI, Table 4.

**Table 2:** Road-traffic injury-related head injury from Nigeria: in-hospital symptomatology of the patients.

Symptoms *N* (%)		Signs *N* (%)	
Loss of consciousness	775 (93.0)	Tachycardia (pulse > 100)	385 (46.2)
ENT effluxes	325 (39.0)	Fever (temperature > 38.5)	218 (26.5)
Vomiting	201 (24.1)	Hypotension (Syst BP < 90)	23 (2.8)
Seizures	109 (13.1)	Anemia (packed cell volume < 30)	156 (18.7)
Headache)	101 (12.1)	Anisocoria	265 (31.8)

**Table 3:** Road-traffic injury-related head injury from Nigeria: distribution of associated extracranial injuries.

Systemic injuries	*N* (%)
Maxillofacial	365 (43.8)
Spine	60 (7.2)
Cervical spine	49 (5.9)
Thoracic spine	9 (1.1)
Lumbosacral	2 (0.2)
Chest	70 (8.4)
Abdomen	26 (3.1)
Pelvis/long bones	173 (20.8)
Pelvis	29 (3.5)
Closed long bone fractures	79 (9.5)
Simple open fractures	47 (5.6)
Complex open fractures	18 (2.2)

**Table 4:** Road-traffic injury-Related head injury (HI) from Nigeria: brain and systemic trauma severity categorization.

Brain/injury severity	Proportion
**Severity of HI using the GCS**	
Mild (GCS 13–15)	415 (49.8)
Moderate (GCS 9–12)	230 (27.6)
Severe (GCS 3–8)	188 (22.6)

**AIS_Head**	
1–3	221 (26.5)
>3	612 (73.5)

**Brain CT Rotterdam score (*N* = 584)**	
1–2	288 (49.3)
≥3	296 (50.7)

**Injury Severity Score (ISS)**	
Median	26.0 (IQR 17–34)
ISS 1–25	412 (49.5)
ISS > 25	421 (50.5)

*Numbers in parentheses are % unless otherwise stated*.

*AIS, Abbreviated Injury Scale (version 1990); CT, computed tomography; GCS, Glasgow Coma Scale; HI, head injury; IQR, interquartile range*.

### Radiological Findings

Cranial CT scan was obtained in 584 (72.2%) of the cases. In the rest of the patients, either there was no clinical indication for brain CT or they could not afford this imaging study. For those who underwent the imaging study, the brain CT revealed possible surgical lesions like extensive brain contusions in 157 (18.8%); acute extradural hematoma in 34 (4.1%); acute subdural hematoma in 32 (3.8%); traumatic intracerebral hemorrhage in 27 (3.2%); and pneumocephalus in 8 (1.0%). The CT Rott score ranged from 1 to 2 in 288 (49.3%) and ≥3 in 296 (50.7%).

### In-Hospital Course/Outcome

Full data were available regarding the in-hospital care received for 757 patients (91%, 757/833). Six hundred and sixty cases (78%) were managed non-operatively while 97 (11.6%) received operative care. Many other surgical candidates died before surgery or had no funds for this undertaking. The in-hospital outcome data were available for 773 (93%) of the patients. Good outcome (GOS: normal–moderate deficit) was recorded in 551/773 (71.3%); poor outcome (GOS: severe deficit-death) in 222/773 (28.7%). Statistical analysis, using the chi-squared test of associations, for the determinants of this outcome profile showed that subjects with worse outcome had significantly more severe HI/trauma (*p* < 0.001) in each case, on the GCS, the CT Rott score, and the ISS Table 5.

**Table 5:** Road-traffic injury-related head injury (HI) from Nigeria: in-hospital outcome and determinants.

Severity of HI using Glasgow Coma Scale (GCS)	*N* (%)	In-hospital outcome good	In-hospital outcome poor	Proportion of death	Proportion of Injury Severity Score > 25 (%)	Proportion CTRot > 3 (%)
Mild HI (GCS 13–15)	415 (49.8)	372 (94.9)	20 (5.1)	16 (3.9)	29.6	7.5
Moderate HI (GCS 9–12)	230 (27.6)	143 (69.1)	64 (30.9)	48 (20.9)	61.3	24.9
Severe HI (GCS 3–8)	188 (22.6)	36 (20.7)*	138 (79.3)*	123 (65.4)*	83.5*	40.8*

*p-value < 0.001 (using the chi-squared analysis of associations) in each case.

## Discussion

This study is a descriptive cross-sectional analysis of all-terrain traffic-related HI managed in a university hospital in Nigeria. It was a retrospective analysis of a prospective HI registry. Road accidents accounted for more than 80% of all HI in this registry. Motorcycles and motor vehicles, the former the more frequent, were the only offending agents involved. The victims, VRU in about three-quarters, were mostly young males, the 20–40 years-of-age group, and were predominantly in the low socioeconomic class. One-third was pedestrians. Significant proportions suffered moderate-severe HI; severe brain injury on imaging, about one-fifth of this being surgical; and associated significant severe systemic injury. In-hospital poor outcome occurred in more than a quarter, and was associated with the severity of the systemic and brain trauma. These are notable findings concerning the clinical epidemiology of RTI-related HI from a developing country in the current era. Some of them are actually unique to this study.

For one, it is the only one that we are aware of that specifically reports all-terrain, RTI-related HI in our region of the world and our findings demonstrate that injuries resulting from road crashes in this part of the world are toward the more severe end of the trauma spectrum. This fact is fully in keeping with the literature evidence that traffic-related fatalities are most prevalent in the developing countries in general, and the African region in particular (1, 7–9, 21). Hence, apart from the usual severe brain injury necessitating their neurosurgical referral in this study subjects, there was usually evidence of significant systemic injuries in the other body parts, especially the limbs and the facial regions. These findings suggest that there is an unfulfilled need for regional multi-disciplinary trauma centers in Nigeria.

Most of the victims were young males in their most productive prime of life, the economic life-wires of most homes in sub-Saharan Africa. Most are from the low-income economic strata of the population, a group well-documented to bear the greatest proportion of the global RTI burden (8, 10, 13, 14, 22, 23). This fact has well-known significant debilitating implications for a nation’s human capital base. It has been estimated to cost a country 1–2% of her gross national productivity per annum as a result of trauma deaths, disabilities and property expenses (1, 8, 24). These deaths also cause unquantifiable personal costs for families including loss of the family bread winner and costs associated with prolonged medical care and the long-term care of the disabled resulting in a high burden of disability adjusted life years in these regions of the world (8, 24). At the more personal level, such huge socioeconomic costs, borne directly by the already impoverished individual victims and his relations in the challenging privately funded health systems of most LMICs actually push many families into poverty in the developing countries (8, 25).

Motor vehicles (cars or buses), motorcycles, and tricycles are currently the main movers of people and goods intra- and inter-city in Nigeria. This study demonstrated that only motorized two- and four-wheeled vehicles were involved in RTI-related HI, with no cases resulting from tricycles, rail, nor bicycles. And from all the RTI-related HI, two-thirds of those injured were VRU and one-third was pedestrians (7). These findings could perhaps give some insights into what locally appropriate interventions may be called for in the efforts to promote a de-escalation of the extant regional staggering burdens of RTIs.

Most times, measures that had proven effectual at promoting road safety and prevention or mitigation of RTI burdens in the high-income countries fail to have any significant impact in the LMICs. Some of these measures include graduated driver licensing system, restrictions on the engine size of motorcycles that learners could ride, raising the legal age of motorcyclists, and construction of overpasses along selected roads and highways. These policies are aimed at reducing the risk of dangerous road scenarios for the vulnerable. Most of them are, however, hardly taken up in the LMICs. Passengers from some developing countries have been known to avoid using overpasses across highways, considering them too long a distance to cover across the highways, and to be high-risk settings for personal crimes (8).

Matter of fact, while striking reduction in the rates of RTI-related fatalities have been noted in the high-income countries, the reverse is the case in the LMICs. A World Bank report actually noted a more than 200% surge in the case fatalities for some of these nations (1, 8). It then became apparent that most of these preventive measures that worked so well in the industrially developed countries might need to be re-cast for the LMICs. The policies appear to not be so beneficial to the VRU, pedestrians and motorcycle victims of RTI in particular, and these groups usually constitute a significant proportion for the RTI burdens in these settings. They represent close to three-quarters (74%) of the trauma burden in this study.

More precisely speaking, therefore, the findings of our study suggest that efforts at reducing the RTI burdens in Nigeria must at least start by addressing the place, the preeminence, the propriety, and the priority of low-capacity motor vehicles as well as motorcycles as the means of mass transportation in this country.

### Limitations of the Study

This study might be biased against less severe forms of HI: the database for it was acquired in a university teaching hospital, actually the nation’s foremost neurosurgical unit, with wide-ranging referral base (16). Also, the study emanated from a single-physician (Neurosurgeon) database in a tertiary hospital. And although the database was a prospective registry, it suffered from the established limitations of the retrospective nature of this particular analysis. It could be argued, however, that the study subjects could actually only be acquired in this sort of practice.

### Conclusion

Road-traffic injury (RTI)-related HI are usually severe, and are associated with significant extracranial injuries in Nigeria. The victims are mostly males in the prime of life. Motor vehicles and motorcycles are the exclusive causation, most frequently in VRU. This revelation offers a most actionable opportunity of an easily identified target for preventive measures.

## Ethical Statement

The directorate of clinical services, research and training of the University College Hospital, UCH, Ibadan, Nigeria gave formal, written institutional approval for the academic audit of the paper-based clinical records used in this study. (Document attached as supplementary file to this manuscript.) The study did not involve any human subjects directly or indirectly.

## Author Contributions

AA conceived the study, managed the study subjects in-hospital, personally involved in data gathering and analysis, drafted the manuscript, and approved the final version for submission. MO helped in data gathering, data analysis, and manuscript drafts. Read and approved the final version for submission.

## Conflict of Interest Statement

The authors declare that the research was conducted in the absence of any commercial or financial relationships that could be construed as a potential conflict of interest. The reviewers SN and handling editor declared their shared affiliation.
