# Supplementary figures and images for: Clinical Epidemiology of Head Injury from Road-Traffic Trauma in a Developing Country in the Current Era
Source: Front Neurol. 2017 Dec 15;8:695. doi: 10.3389/fneur.2017.00695 (PMC5736536; doi:10.3389/fneur.2017.00695)

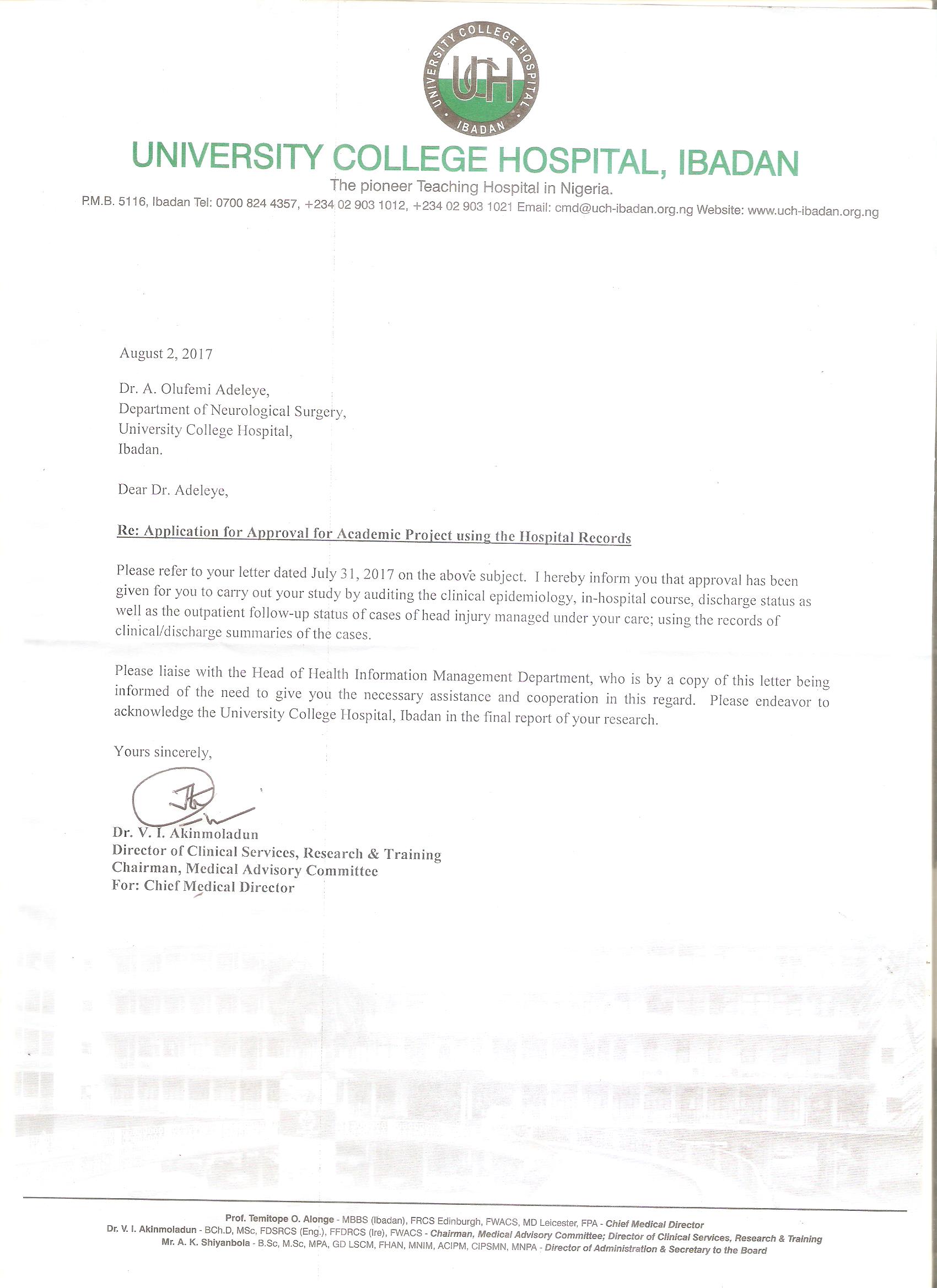

Supplement: Supplementary file 1 [file Image_1.jpg]
